# The effect of cartilage and bone density of mushroom-shaped, photooxidized, osteochondral transplants: an experimental study on graft performance in sheep using transplants originating from different species

**DOI:** 10.1186/1471-2474-6-60

**Published:** 2005-12-15

**Authors:** Anja C Waselau, Daniel Nadler, Jessika MV Müller, Katalin Zlinszky, Monika Hilbe, Jörg A Auer, Brigitte von Rechenberg

**Affiliations:** 1 Musculoskeletal Research Unit, Equine Hospital, Vetsuisse Faculty Zurich, University of Zurich, Switzerland; 2 Anesthesiology, Equine Hospital, Vetsuisse Faculty Zurich, University of Zurich, Switzerland; 3 Veterinary Pathology, Vetsuisse Faculty Zurich, University of Zurich, Switzerland; 4 Centerpulse Biologics, Winterthur, Switzerland

## Abstract

**Background:**

Differences in overall performance of osteochondral photooxidized grafts were studied in accordance of their species origin and a new, more rigorous cleansing procedure using alcohol during preparation.

**Methods:**

Photooxidized mushroom-shaped grafts of bovine, ovine, human and equine origin were implanted in the femoral condyles of 32 sheep (condyles: n = 64). No viable chondrocytes were present at the time of implantation. Grafts were evaluated at 6 months using plastic embedded sections of non-decalcified bone and cartilage specimens. Graft incorporation, the formation of cyst-like lesions at the base of the cartilage junction as well as cartilage morphology was studied qualitatively, semi-quantitatively using a score system and quantitatively by performing histomorphometrical measurements of percentage of bone and fibrous tissue of the original defects. For statistical analysis a factorial analysis of variance (ANOVA- test) was applied.

**Results:**

Differences of graft performance were found according to species origin and cleansing process during graft preparation. According to the score system cartilage surface integrity was best for equine grafts, as well as dislocation or mechanical stability. The equine grafts showed the highest percentage for bone and lowest for fibrous tissue, resp. cystic lesions. The new, more rigorous cleansing process decreased cartilage persistence and overall graft performance.

**Conclusion:**

Performance of grafts from equine origin was better compared to bovine, ovine and human grafts. The exact reason for this difference was not proven in the current study, but could be related to differences in density of cartilage and subchondral bone between species.

## Background

Osteochondral transplants for cartilage resurfacing have been used with variable success either as single autografts[1], or in mosaicplasty[2]. Problems such as donor morbidity at the harvesting site and invading a second joint for harvesting lead to the search of pretreated, photooxidized transplants that were successfully applied in experimental sheep either as cylinders[3,4] or mushroom shaped grafts[5] into the femoral condyles. These studies revealed that the density of the subchondral bone of the grafts and the host play a pivotal role in graft stability and overall survival as well as the process of photooxidation [4,5]. There, photooxidized, mushroom-shaped, bovine osteochondral transplants performed superior in articular cartilage resurfacing in short and long-term experiments in sheep compared to the less dense autografts or ovine allografts[3,4]. The higher density and the photooxidation process were shown to slow down resorption of the graft. This resistance to resorption was held responsible for improved osseointegration of the grafts preventing their premature collapse into the original defect.

Photooxidation, a process based on immersion of tissue in methylene blue under exposure to a halogen light source, has been used to stabilize biological tissue for surgical implantation[3], and was shown to serve well as scaffold for tissue-guided regeneration, at least in osteochondral cartilage transplants [3-5]. Pretreatment with photooxidation reduced antigenicity of the xenografts by changing the fine structure of the collagen fibres [6] and left no viable chondrocytes within the cartilage matrix. No immunologic reactions were noticed histologically [4,5], in contrast to experiments where untreated porcine and bovine osteochondral plugs were implanted into the suprapatellar pouches of the stifle joint evoking strong immunogenic reactions in monkeys [7]. This was also partially true for pretreatment of xenografts with lyophilization [8], where immunogenicity was reduced, but chondrocyte viability was partially maintained [9]. Although after photooxidation no living cells were visible in histological sections, partial repopulation with cells was already recorded at 2, 6, and almost complete repopulation at 12 and 18 months when bovine, cylindrical and/or mushroom grafts were inserted into medial and lateral femoral condyles of sheep [4,5].

Apart from mechanical stability of the grafts [10,11], joint congruency was considered very important [12], as well as the preservation of the subchondral bone [4,5]. The development of cystic lesions was noticed at the interface between graft and host bone with fresh, sternal cartilage transplants in horses [13] and photooxidized, cylindrical grafts in sheep [3]. In the latter, the peak of cyst development was at 6 months after implantation [3] and subsided mostly at 12 months, if grafts were mechanically stable and not dislocated [4]. The mushroom structure reduced cyst formation considerably compared to cylindrical grafts.

The goal of this experiment was *i) *to study the effect on species differences by comparing photooxidized, mushroom-shaped bovine, human and ovine grafts, in addition to *ii) *investigating the effect of a new cleansing procedure of the photooxidized grafts. Equine grafts were also included in the groups with the new cleansing procedure. The rationale of the study was based on the assumption that a transplant with a higher cartilage and bone density would improve overall performance, while a more rigorous cleansing process would reduce the immunogenicity and hopefully the speed of resorption, especially in the bony part of the transplants. According to the bone density, it was expected that equine grafts would best fulfill these requirements.

## Methods

### Experimental animals

Thirty two (32) female, Swiss Alpine sheep with a mean age of 2.5 years (range 2–3 years) and 70 kg of body weight (range 48–91 kg) served as experimental animals. Prior to surgery the animals were adapted to the new stable environment for 14 days, checked for their overall health (physical exam and blood check), dewormed (Cestocur^®^; Bayer AG, Leverkusen, Germany; Endex^®^; Novartis AG; Bale; Switzerland), vaccinated against Clostridium difficile and tetanus (Pulpyvax^®^-T s.c.; Essex Veterianaria AG; Bern, Switzerland) and their claws were inspected. The sheep were randomly assigned to 6 groups with 4 animals each and 1 group with 8 animals according to the distribution of the osteochondral grafts *[see *Additional file 1*]*. In each animal, independent of group distribution, 2 grafts of the same origin and treatment were implanted into the weight bearing area of the distal medial and lateral femoral condyle alternating between right and left limbs. Thus, a total of 64 osteochondral grafts were implanted. All animal experiments were conducted according to the national laws of animal welfare and protection with the permission of the local Ethical Committee and official veterinary authorities (# 08/2002).

### Osteochondral transplants

Mushroom-structured, photooxidized osteochondral grafts were used as transplants originating from different species. Equine (EN) and bovine (BO, BN) grafts were harvested from shoulder joints of healthy animals (age: 10–18 months for heifers, between 2 – 5 years of age for horses) in the local slaughterhouse within 12 hours after death of the animals, while ovine grafts (OO,ON) were taken at the university facilities immediately after sacrifice of experimental sheep (age between 2–3 years) killed for other reasons than infectious or joint disease. While bovine, ovine and equine grafts were collected at the slaughterhouse, the human grafts were received from a registered bone bank and treated with the photooxidation process using the exact same protocol. Graft harvesting and preparation was performed as described before [3-5]. Briefly, cylindrical osteochondral plugs were harvested from the weight bearing area of the proximal humerus using a diamond coated drill bit (Draenert, Munich, Germany). Before harvesting, the cartilage surface was assessed macroscopically for absence of signs of cartilage degradation, such as fibrillation, discoloration and loss of shiny appearance. The cartilage plugs were then further processed through an automated process resulting in mushroom-shaped grafts with a 6 mm overall diameter of the mushroom head and a 3 mm width of the mushroom stem. The overall length of the grafts was 7 mm divided in 3 mm length of the mushroom head including the calcified cartilage zone and a minimal amount of subchondral bone, and a 4 mm long stem. Thereafter, the osteochondral mushroom-structured grafts were photooxidized, where a cleansing procedure was followed by a prolonged bathing process in 0.01% methylene blue solution in buffered saline while exposure to a halogen light source took place under stable room temperature conditions (*patent # EP0768332A1, US5,817,153*) [6]. Harvesting and preparation of the grafts were performed under sterile conditions. Grafts were lyophilized and kept under sterile conditions before surgical implantation. The original process was applied for bovine, ovine and human grafts (BO,OO,HO). For the other groups (BN, EN, HN and ON), the cleansing process was intensified, such that a more rigorous washing procedure with alcohol was used compared to the other 3 original groups (BO, OO, HO). Equine grafts were prepared only with the new cleansing procedure.

### Animal anesthesia and surgery

The sheep were routinely prepared for surgery and operated as described before [4,5]. Twentyfour (24) hours prior to surgery the animals were fasted, the surgery field clipped and cleansed. Anesthesia was induced with ketamine hydrochloride (2 mg/kg bw. i.v., Narketan, Chassot AG, Switzerland) in combination with valium (0.01 mg/kg bw., Diazepam, Roche, Switzerland) after sedating the sheep with medetomidine (5 μg/kg bw., im., Domitor, Orion-Farmos, Turku, Finland). Anesthesia was maintained with isoflurane (Forene, Abbott, AG, Baar, Switzerland) in 100% oxygen using endotracheal intubation.

**Figure 1:**
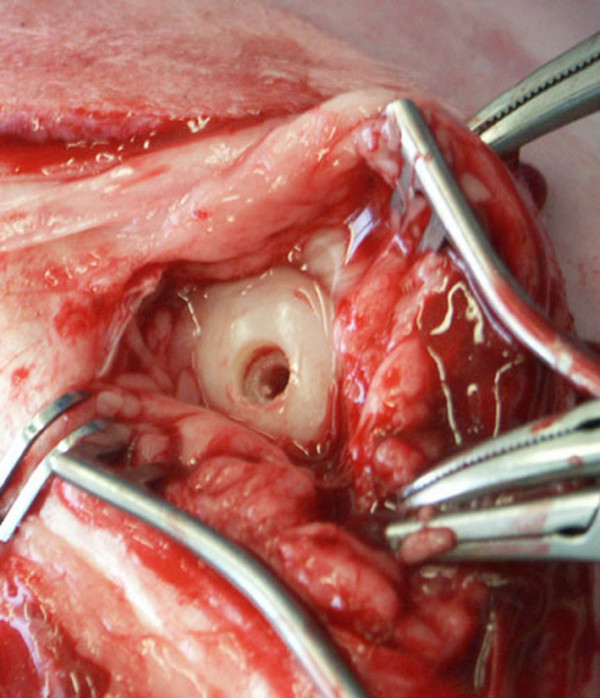
Intraoperative picture of prepared host bed. Note that the drill hole for the mushroom head extends just into the subchondral bone area.

**Figure 2:**
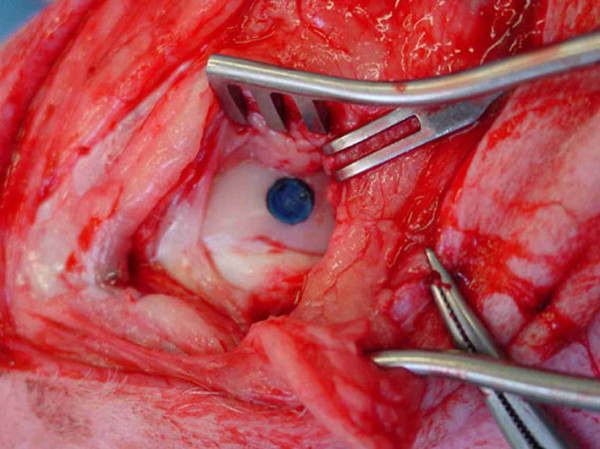
Photooxidized mushroom shaped transplant *in situ *after insertion according to the press fit technique.

A parapatellar approach to the lateral and medial condyles, with the animals in lateral recumbency and the surgery limb in supine and maximally bent position was chosen. With a specially designed drill bit [5] (Centerpulse Biologics, Winterthur, Switzerland) the bed for the mushroom-shaped grafts was prepared in the weight bearing area of both condyles (Fig. 1). The grafts were applied according to the pressfit technique[14,15], where the cartilage surface was left flush with the adjacent cartilage and the stem and the mushroom head were stable within the host bed (Fig. 2). The wound closure was routine.

After surgery the sheep were kept in small stalls for 2 weeks, thereafter released to a larger pen for another 4 weeks and subsequently allowed to roam freely on the pasture throughout the rest of the study. At 6 months after surgery all animals were sacrificed and the stifle joints immediately harvested.

### Evaluation of samples

The stifle joints were carefully opened, the patellar ligaments severed and reflected dorsally and the joint capsule was evaluated macroscopically for signs of inflammation, such as reddening of the tissue, hypertrophy of the villous part of the synovial membrane, tissue adhesions, appearance of the fat pad and color of the synovial fluid. The cartilage surface was studied for position, surface and staining of the original graft, graft-host cartilage interface as well as the surface of the host cartilage. Obvious signs of matrix degradation, such as fibrillation, cleft formation, cobble stone appearance and discoloration of the hyaline cartilage surface were recorded and compared among groups. All samples were documented using a digital camera (Minolta^® ^Dimage7).

### Sample preparation

The stifle joints were then further prepared for sampling of probes such, that synovial membrane samples were harvested within the villous part immediately cranial to the lateral and medial aspects of the proximal tibia plateau. Then the femoral condyles were freed from the soft tissues and 1 × 1 cm blocks containing the original grafts were cut using a bone saw (K 410, Kolbe GmbH, Elchingen, Germany).

### Radiology

The bone blocks were radiographed using a faxitron (Cabinet x-ray-faxitron series, model 43855A, Hewlett Packard^®^, USA) with Fuji Photo Films (Fuji Photo Film Co.^®^, Ltd, Tokyo, Japan) from a cranio-caudal and latero-medial view before further processing.

### Histology

The bone and synovial membrane samples were fixed in 4% paraformaldehyde at 4°C and further processed for histology. The synovial membrane samples were prepared for routine histology using paraffin blocks, where sections were stained with hematoxilin eosine (HE). Bone samples were embedded in acrylic resin based on methyl methacrylate as described before [4,5,16,17]. Briefly, the bone samples were fixed for ca. 5–7 days, washed in phosphate buffered saline (PBS), before they were dehydrated in a series of alcohol, defatted in xylene, infiltrated with methyl methacrylate (methacrylacid-methylester; dibuthylphtalate and perkadox in a proportion 89,5: 10: 0,5) and then embedded in the same solution using special Teflon molds (custom made in-house by Centerpulse) that were placed in a water bath at 30°C. Ground (30–40 μm) and thin (5 μm) sections were cut (Leica^® ^SP 1600 and Leica^® ^RM 2155; Leica Instruments GmbH, Nussloch, Germany). Ground sections were surface stained with toluidine blue, while thin sections were deplastified with Methoxyethyl-acetate (Merck, Schweiz AG) and stained with toluidine blue or von Kossa/McNeal. Toluidine blue allows assessing metachromatic staining of cartilage matrix due to proteoglycan content, while the staining method according to von Kossa /McNeal distinguishes between calcified bone matrix and non-mineralized osteoid. Furthermore, cytoplasms of cells are stained turquoise blue.

### Evaluation of histology sections

Synovial membranes were evaluated for signs of immunological reactions and inflammations through 3 independent reviewers. A semi-quantitative score system was developed for morphology and proliferation of synoviocytes, appearance of neutrophilic and eosinophilic granulocytes, lymphocytes, plasma cells and macrophages. The neoformation of capillaries and small vessels, amount of fibrin exsudate and metaplasia of fibrous tissue was also assessed. The score system for each variable is depicted in *[see *Additional file 2*]*. If the assessment of the reviewers differed, the mean value was used for further statistical analysis.

Cartilage repair was evaluated qualitatively, semi-quantitatively and by means of histomorphometry. In the *qualitative evaluation *by means of a light microscope (Leica, DMR, Glattbrugg, Switzerland) focus was placed on cell types involved in mechanisms of cartilage repair, viability of cell grafts, quality of interface between host and grafts, as well as subchondral bone remodeling and cyst development. For *semi-quantitative evaluation *a score system was developed that was based on a previously published system by our research group [4,5]. Scores were divided in regenerative and degenerative aspects ranging between 0–3, where low scores represented good and high scores bad results *[see *Additional file 3*]*. Two reviewers evaluated the sections independently, before scores were compared. If differences occurred, mean values were used for final statistical analysis. For *quantitative evaluation *by means of histomorphometry ground sections were used that were captured through a macroscope (Leica, M420, Glattbrugg, Switzerland) as digital images in TIF-format (DC 200, Leica. Glattbrugg, Switzerland) and at a 5.8 times magnification. As reported in an earlier study with those type of grafts, the digitalized pictures were colored before measuring for better detection of the fractions cartilage matrix, bone, fibrous tissue, resp. bone marrow [5]. Using a special software program (Leica Qwin^®^, Leica Quips^®^) the areas of the different fractions were measured in μm and the percentage of each was calculated.

### Statistical analysis

Results were analyzed using a factorial analysis of variance (ANOVA, StatView^®^, Version 4.5, Abacus concepts, California, USA) to assess overall differences between the various groups. Specific differences between individual groups were calculated using post hoc Scheffé tests. Significance was set at p-values < 0.05.

## Results

*Graft preparation *with the automated process went uneventfully resulting in even consistency and appearance for the bovine, ovine and equine groups. Preparation of human grafts was slightly more variable, such that the cartilage surface seemed less elastic and differences in cartilage thickness, density and evenness of surface structure were recorded (groups HN and HO). Other species differences were noticed, such that density of the subchondral bone area was different. The equine showed the highest density followed by bovine, ovine and last human grafts.

*Surgery *was performed successfully in all 64 grafts (32 lateral and 32 medial condyles, animals:n = 32). In most of the animals the grafts fitted well into the host bed. However, due to slight differences between the cartilage thickness of the grafts and the adjacent host, minimal incongruencies (± 0.1 – 0.3 mm) could not always be avoided, especially in the groups with human grafts.

### Clinical observations

The animals were slightly lame for maximally 10 days after implantation, but, thereafter, lameness disappeared in all of the animals for the rest of the study period. In one animal of the group with the ovine grafts (ON) diffuse swelling of the stifle joint without clinical lameness was found at 3 weeks after surgery lasting only for about 5 days before normal joint filling was resumed.

**Figure 3:**
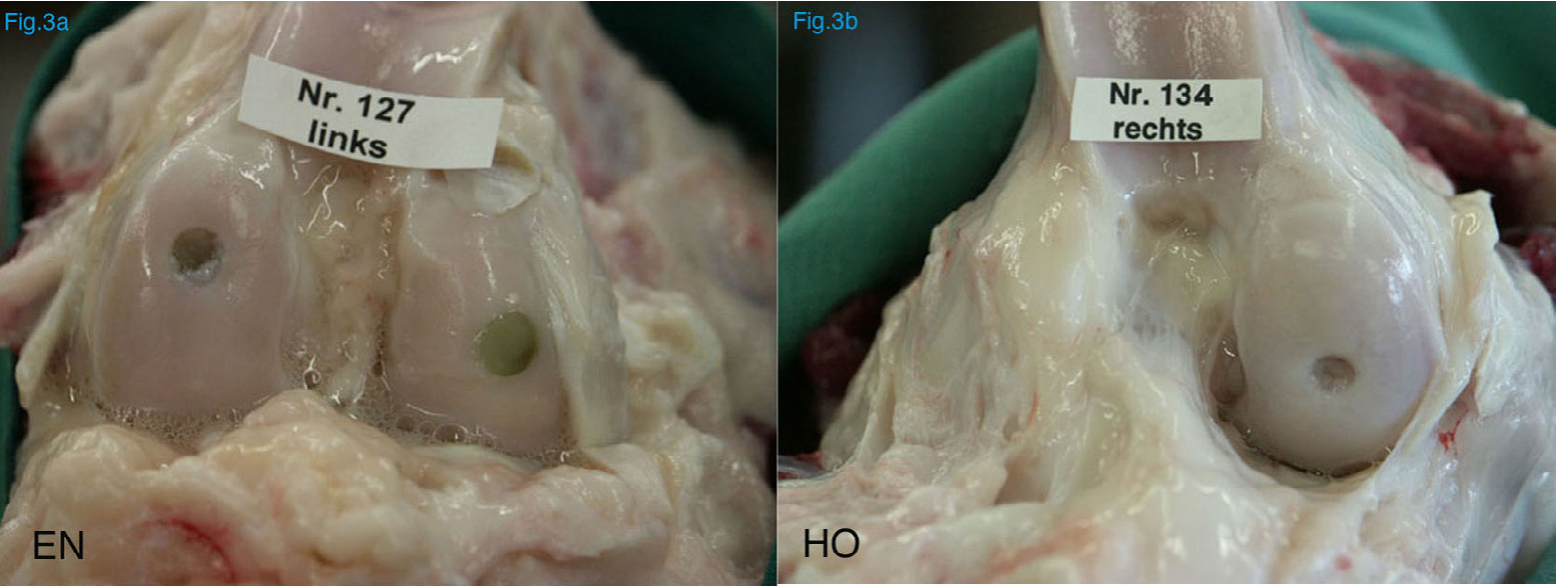
Macroscopic evaluation of the stifle joints after insertion of a mushroom shaped transplant of equine (*EN*) and human (*HO*) origin. While the soft tissue structures appeared macroscopically normal with the equine grafts, major adhesions were found in two specimens with the human grafts (*HO*).

*Macroscopical evaluation *at the time of sacrifice at 6 months revealed absence of joint inflammation, except in 2 joints of the human grafts (HO) where adhesions between the joint capsule, fat pad and collateral joint compartment were found (Fig. 3). Furthermore, signs of mild synovitis were found in all joints where human grafts were implanted (groups HN and HO).

**Figure 4:**
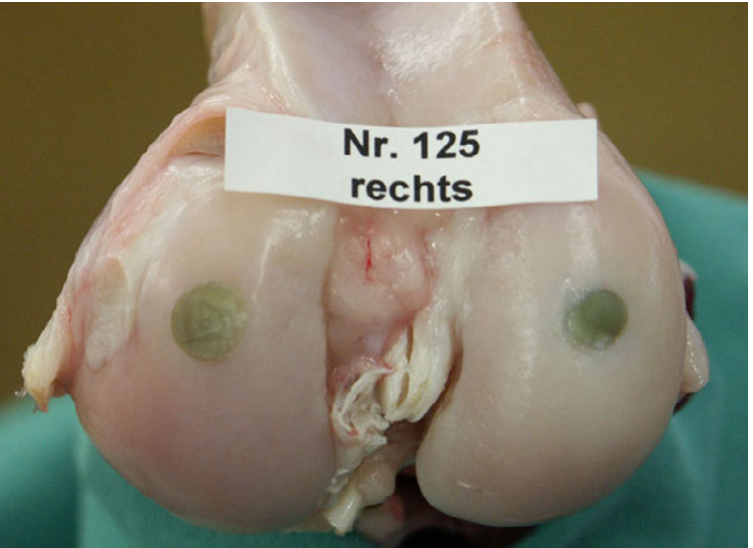
The bluish color was still present in most of the grafts at the time of sacrifice (6 months). A small seam of cartilage tissue was visible at the periphery of the graft stemming from the surrounding cartilage and growing towards the center of the grafts (*EN*).

All grafts were still in their original location, but positioning differed between species. Overall, 24 of the total 64 grafts were in excellent position and 40 grafts were sunk slightly into the original defect *[see *Additional file 4*]*. The bluish color of the original graft was still visible in 50, whereas it had almost disappeared in 14 of the condyles (Fig. 4). A pannus-like type of tissue was observed growing from the periphery towards the center of the defect in 33 of the condyles. Small osteophytes were detected in 2 medial condyles of the BN, and 2 of the HO group. The animals of the HO group were the same where the adhesions were found, while no obvious explanation could be found for the 2 animals of the BN group. As an overall impression, the equine grafts (EN) showed the best macroscopic results such, that only 1 of 8 implants (12.5%) was sunk into the original defect and all other grafts maintained their original position. The bluish color was still visible in all equine grafts and only in 2 grafts a small seam of cartilage-like tissue was noted at the periphery.

*Radiographic evaluation *revealed cystic lesions, either within the original defect and/or extending into the adjacent bone *[see *Additional file 5*]*. Out of 64 transplants 28 cystic lesions were found. The highest percentage was seen in the HO group (100%), followed by BN and BO with 50% each, and in all other groups only 25% were affected. The size of the cysts was not specifically measured.

### Histological evaluation

**Figure 5:**
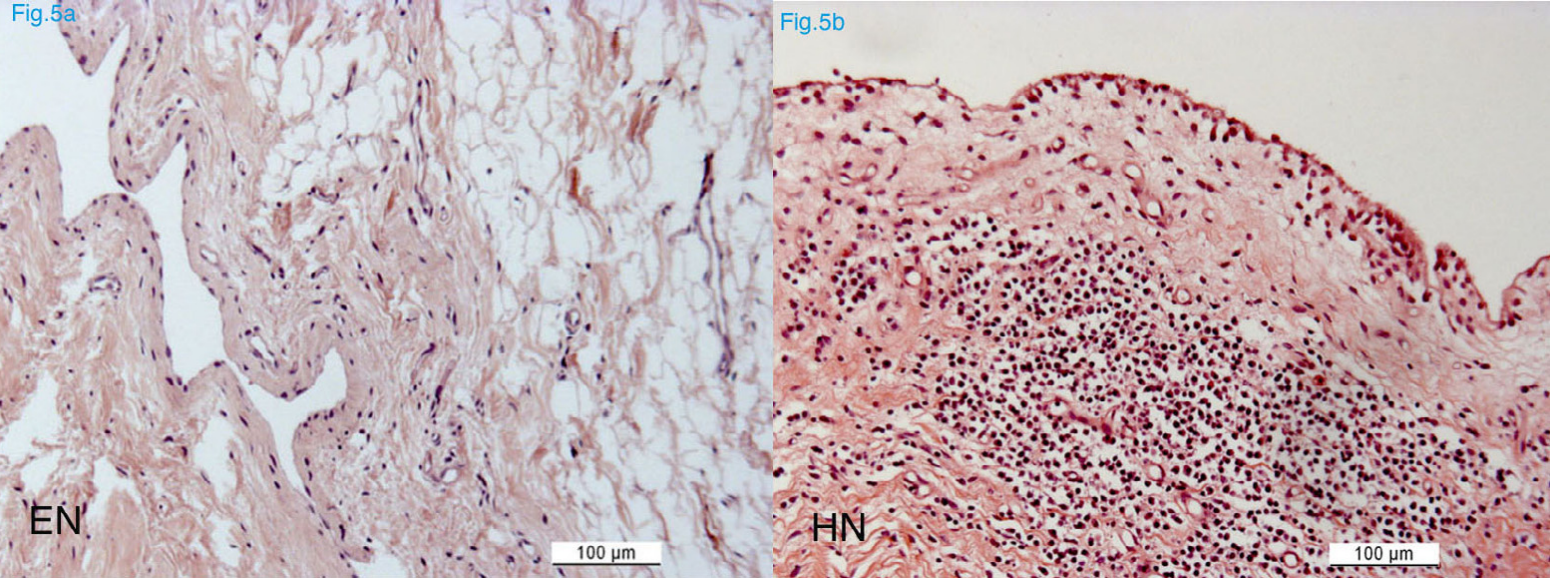
Histological sections of synovial membranes of the equine (*EN*) and the human group (*HN*) (*paraffin sections, HE staining*). Signs of inflammation were recorded indicated by infiltration with mononuclear cells and neutrophils.

Histological assessment confirmed the macroscopical findings. Mild immunological reactions of the synovial membranes could only be detected in the groups with the human grafts (HN and HO), but never with the other species (Fig. 5). Although, the scores differed significantly for synovial membranes, the findings were not pathological in all specimens.

**Figure 6:**
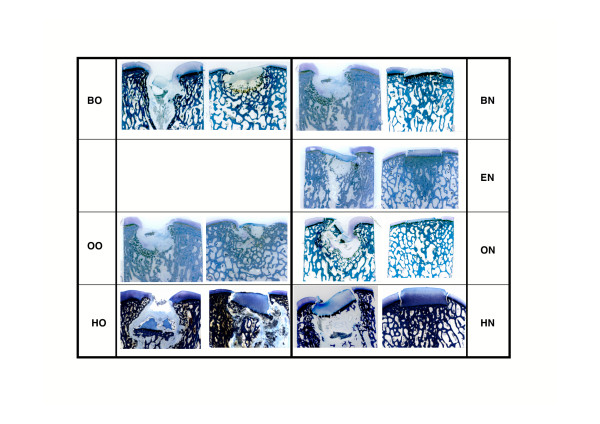
Overview of ground sections (30–40 μm, toluidine blue) from all groups. Variability between and within groups was quite high. In each group, the left sample represents the worst, and the right the best performing transplants.

**Figure 7:**
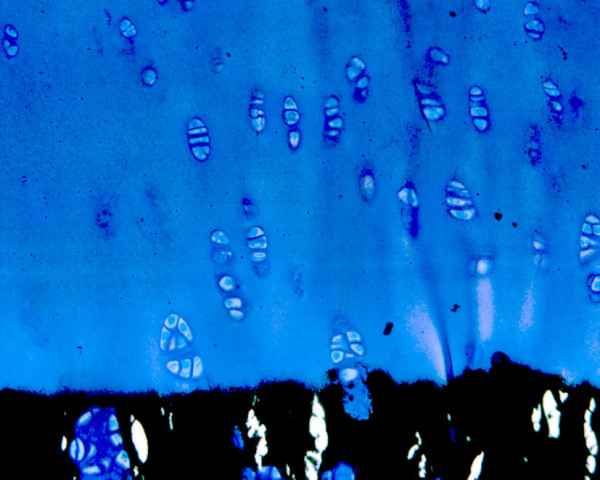
Repopulation with new chondroid cells was noticed in the deep cartilage zone of the grafts in most of the specimens after 6 months shown by a dark blue periterritorial zone around the chondrocytes indicating new proteoglycan synthesis (5 μm section of an equine (EN) graft, von Kossa/McNeal).

The cartilage surface varied between species with the equine grafts showing an almost intact photooxidized cartilage matrix in all specimens. Fusion between host and graft cartilage was rarely observed at 6 months and only at the junction of the calcified cartilage layer and the subchondral bone. Creeping substitution of the graft bone by new bone formation from the host was visible in all sections. The new bone could be easily distinguished from the original (dead) graft bone through its woven structure and darker blue color. The original graft exhibited still its original lamellar structure and its lighter blue coloration. Even if the original graft was not replaced, infiltration with osteoblast progenitor cells and deposition of new osteoid at the surface of the trabecula was noticed also in the center of the grafts. If grafts maintained their original position, the integration of the subchondral bone was excellent in all grafts independent of the species (Fig. 6). In those grafts dislocated into the original defect, increased cyst formation could be noticed. However, even in those grafts the integration and remodeling of the subchondral bone was in progress. Repopulation of the photooxidized matrix with viable cells was seen only in the deep zone close to the calcified cartilage zone and the tide mark (Fig. 7).

### Semi-quantitative evaluation

The assessment of all independent reviewers differed only minimally, indicating that the results were relatively clear. That was true for both, the synovial membrane and the cartilage.

Histological changes of synovial membranes were minimal for most groups. Only mild signs of inflammation were noticed mostly with the human grafts (HO, HN). The synovial membranes *[see *Additional file 6*] *demonstrated excellent scores ( = 0) for the equine grafts (EN), followed closely by the groups of the bovine old and ovine old (BO, OO). The differences were statistically significant to all other groups (P < 0.05).

The evaluation of the cartilage scores was more difficult and complex and are outlined in *tab.7 [see *Additional file 7*]*. Since the best scores for histological variables were not always contained within the same group of transplants, the following selection was made for best overall performance of the grafts. The highest, the second best and the lowest scores were chosen for each histological variable. Finally, the group with the accumulation of most of the best and second best scores was considered superior to the other groups.

Like with the synovial membrane, the equine grafts revealed the best overall results also for the cartilage. Most of the best scores (total = 5) were found for the equine group (EN) with cartilage surface (0.44 ± 0.5), dislocation of graft (0.36 ± 0.9), cartilage viability of the host (0.25 ± 0.25), chondrocyte proliferation in the host cartilage (0.5 ± 0.5), and cluster formation in the host (0.75 ± 0.7). Most of the second best scores (total = 4) were also found in the equine group with fibrillation of matrix (0.25 ± 0.5), cleft formation on matrix (0.5 ± 0.73), cartilage viability in graft (1.38 ± 0.5), and remodeling of the tide line/calcified cartilage zone (0.63 ± 1.1). Three of the best scores were found in the groups of the bovine old (BO), human new (HN) and ovine old (OO), but three of the second best scores were only found in the human new (HN) and ovine old (OO). Most of the lowest histology scores (total = 7) were found in the group HO.

### Histomorphometry

**Figure 8:**
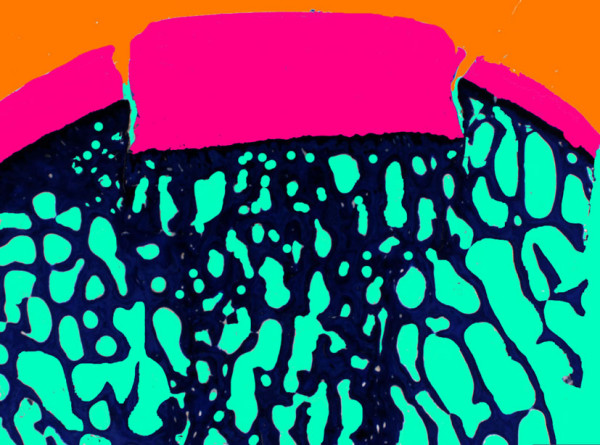
Sample of colored digital picture as it was used for histomorphometrical measurements. Digital images of ground sections (30–40 μm. toluidine blue, 5.8 times magnification) were used. The sample represents a colored ground section of the equine group (EN).

The coloring of the digital pictures improved the exactness of the measurements considerably, such that repeatable results could be obtained (Fig. 8). Overall significant differences were found for percentage of bone (p = 0.0043) and fibrous tissue (p = 0.0002). Individual differences are outlined in *tab.8 [see *Additional file 8*]*. Measurements of percentage of bone confirmed our semiquantitative evaluation, such that equine grafts showed the highest percentage for bone (51.31 ± 11.1) and lowest for fibrous tissue, resp. cystic lesions (29.87 ± 5.6). The worst results were found for the human old (HO), where the percentage of bone was lowest (30.82 ± 4.6) and highest for fibrous tissue (48.66 ± 11.4).

## Discussion

In this study photooxidized, mushroom shaped, osteochondral grafts of equine origin implanted in sheep were superior in cartilage surface integrity, persistence of grafts and reaction of adjacent host tissue at 6 months after implantation, if compared to equally pretreated bovine, ovine and human grafts. The slower resorption and different cartilage and bone density of the equine grafts was held responsible for their improved overall performance. Additional groups of ovine, human and bovine grafts were subjected to a more intensive cleansing procedure that was shown to reduce the durability of the cartilage surface, but also the subchondral bone. However, if the effect of the species was compared to the cleansing procedure, the cartilage and mainly bone density due to species differences seemed to be more important. The equine grafts showed the best results, even though this group was represented only through grafts that had undergone the additional cleansing procedure.

The human transplants could not be prepared exactly as those of the other species. This was due to official regulatory problems, such that catilage grafts could only be obtained from an official bone bank. This may have been the reason, why these transplants were not of the same and even quality as all other grafts. This was especially true for the cartilage surface that looked macroscopically more hampered, but also for the more uneven thickness of the human cartilage. It may well be that the overall results would have been improved for both groups with human grafts if the same harvesting standard could have been maintained also with the human grafts. The equine grafts (EN) showed the most consistent good quality, although only grafts with the more rigorous cleansing procedure were used.

Surface incongruencies between graft and host cartilage could not always be avoided, although its importance was addressed also by other authors[1,3,12,14,18-22]. Since biological transplants will never be completely symmetrical also in auto- transplantations, a minimal step at the superficial cartilage layer and/or at the calcified cartilage zone and the tidemark will always occur. Although an attempt has to be made to implant the graft flush with the cartilage surface, a difference up to 0.1–0.3 mm has to be acceptable for clinical reality. As long as the grafts are not left proud of the surface[12], this should not seriously jeopardize the outcome, at least with photooxodized grafts [4,5].

Based on our earlier experiments the time point of sacrifice was chosen at 6 months. Cyst formation was most pronounced at this time period and was considered instrumental for graft survival [4,5]. This was confirmed in this study, where cystic lesions were found in about one third of the grafts. The size of the lesions was not measured radiographically or histologically, since both evaluation techniques represent only two-dimensional techniques. The three dimensional aspect could have been included if microcomputer tomography would have been performed [23-26]. However, it is questionable whether it would have influenced the overall results of this study. The authors felt that calculating the percentage of fibrous versus bony tissue by using histomorphometry reflected the situation of cystic lesions adequately enough to draw valid conclusions.

The score system for semi-quantitative assessment of graft performance was the same as used in our previously published studies [4,5]. Although it addressed similar aspects as classic scoring systems for grading cartilage [27,28], it had to be adapted to the specific nature of the grafts. This was especially true in view of the photooxidation process that left an intact matrix as for the structure of the collagen macromolecules, but no living chondrocytes. Cluster formation is considered to be a classic sign of cartilage degeneration [18,29-32] and was handled the same way in our adapted scoring system (*score a = degenerative*). However, this may not be correct. As in early osteoarthritis it may be an attempt of the tissue to prevent matrix degradation [33] and thus, in the case of the photooxidized cartilage actually be a positive sign for viability and repopulation with living cells. Furthermore, each score evaluated may not have the same importance. Thus, the total sum of the score may not truly represent the best graft performance in the overall picture. Surface integrity and graft dislocation are certainly among the most important features for graft survival and there, equine grafts (EN) showed the best results. Nevertheless, as in our previous work, the adapted score system served well its purpose to evaluate graft performance.

Compared to earlier results obtained with photooxidized grafts [3-5] viability and repopulation with new cells of the original photooxidized grafts was relatively low. This was dependent on the implantation period of only 6 months duration in this study, whereas in the previous work graft survival was followed for 12 and 18 months. There, viability, repopulation as well as fusion between host and graft were mainly reported for later time points. Nevertheless, ingrowing cells were noticed also already at 6 months in both studies, but mainly restricted in the deep zone of the cartilage close to the calcified cartilage zone and tidemark. The intensive remodeling of the calcified cartilage including the tide mark suggested that these cells originated from the subchondral bone area.

The intensified cleansing procedure applied on the photooxidized grafts was harmful on structural properties of the grafts. This was already visible macroscopically such, that the cartilage surfaces were roughened at the time of implantation. This may be one of the main reasons, why bovine, ovine and human grafts undergoing this process did not perform at the same high level as their counterpart groups subjected to the original photooxidation process in this study and also in the previously published experiments [4,5,34]. Nevertheless, equine, photooxidized, mushroom-shaped, osteochondral grafts showed the best overall results at 6 months after implantation despite the fact that only grafts prepared with the more rigorous cleansing process were used. This was attributed to the slower resorption of the bony part of the graft and high persistence of the cartilage matrix. The slower bone resorption may have resulted in better mechanical stability and thus, graft incorporation in the subchondral bone of the host. The integrity of the cartilage matrix may be due to the relative high density and strong mechanical properties of equine cartilage [35-37]. Although these features were not measured in the current study, the denser appearance of the equine bone in histology sections and the fact that harvesting the plugs with the same instrumentation resulted in a much higher resistance to the hollow drill bit support this assumption on an empirical level. It could be speculated that equine grafts prepared with the original photooxidation process would have resulted in even better performance compared to this study. In the current study, the main interest was focused on the effect of the species differences and graft resistence after implantation, where equine grafts performed best although only grafts with the more vigorous cleansing procedure were tested. Future studies should test the old photooxidation process also for the equine grafts as well as immunogenicity problems that may be related to using equine grafts for other species.

## Conclusion

Differences were found in the performance of photooxidized, mushroom-shaped osteochondral grafts of bovine, ovine, human and equine origin, if grafts were implanted in the medial and lateral femoral condyles of sheep. The superior performance of the equine grafts was attributed to the higher cartilage and subchondral bone density of the grafts compared to the other species, although in the current study differences in density and mechanical properties were not proven by scientific means such as biomechanical testing or density measurements. The slower resorption of the equine grafts during the process of osteointegration and photooxidized cartilage matrix repopulation seemed to be favorable to overall graft survival and performance, at least after 6 months. The newly applied, more rigorous cleansing process with alcohol was not beneficial for graft survival.

## Competing interests

The author(s) declare that they have no competing interests

## Authors' contributions

ACW was the doctorate student and main investigator in the study

DN was the engineer developing the production of the photooxidized grafts

JM was the anaesthetist for all animal surgeries

KZ was responsible for histology

MH supported ACW in the evaluation of the synovial membrane histology

JA was the main surgeon of the experiments

BvR was the assistant of all surgeries and the senior scientist of the whole study

## Pre-publication history

The pre-publication history for this paper can be accessed here:



## Supplementary Material

Additional File 1 Groups of animals and distribution of osteochondral grafts within the groups. Click here for file

Additional File 2 Semi-quantitative score system for histological assessment of synovial membrane sections. High numbers represent bad, low numbers good results. Click here for file

Additional File 3 Semi-quantitative score system for histological assessment of cartilage sections. High numbers represent bad, low numbers good results. Click here for file

Additional File 4 Results of macroscopic evaluation of grafts at the time of sacrifice (6 months). Overall, the equine (EN) together with the old bovine (BO) grafts retained the blue color the most, and only 2 grafts were partially overgrown by pannus-like tissue. Only 1/8 equine grafts was slightly sunk into the original cartilage defect. Click here for file

Additional File 5 Radiographic evaluation of osteochondral transplants at the time of sacrifice (6 months). Click here for file

Additional File 6 Results of semi-quantitative histological evaluation of synovial membrane samples. Scores for *a*) degenerative aspects: 0 = none, 1 = mild, 2 = moderate, 3 = severe and *b*) regenerative aspects: 0 = good, 1 = medium, 2 = few, 3 = none. Note that most of the membranes are within physiological limits and only those of the human transplants show immunogenic reactions. Click here for file

Additional File 7Overview of statistical results (cartilage and subchondral bone) and results of semi-quantitative histological evaluation of cartilage samples. The equine group (EN) accumulates most of the best ( = lowest scores) and second best scores. Most of the lowest scores were found in the human group according to the old process (HO). Scores for *a*) degenerative aspects: 0 = none, 1 = mild, 2 = moderate, 3 = severe and *b*) regenerative aspects: 0 = good, 1 = medium, 2 = few, 3 = none. Low scores represent good results, while high scores mean less good results. Colors indicate performance  best group  second best group  worst groupClick here for file

Additional File 8 Results of the histomorphometrical measurements. The equine group (EN) showed the highest percentage of bone and the least amount of cystic lesions. Click here for file
