# Supplementary material for: The effect of cartilage and bone density of mushroom-shaped, photooxidized, osteochondral transplants: an experimental study on graft performance in sheep using transplants originating from different species
Source: BMC Musculoskelet Disord. 2005 Dec 15;6:60. doi: 10.1186/1471-2474-6-60 (PMC1343563; doi:10.1186/1471-2474-6-60)
Supplement: Additional File 1 — Groups of animals and distribution of osteochondral grafts within the groups. [file 1471-2474-6-60-S1.pdf]

Tab.1 : Arrangement of groups

Material	Process	Abreviation	Animals	Number of Implants
Bovine	new Process	BN	8	16
	old Process	BO	4	8
Ovine	new Process	ON	4	8
	old Process	OO	4	8
Equine	new Process	EN	4	8
Human	new Process	HN	4	8
	old Process	HO	4	8
Total			32	64
