# Supplementary material for: The effect of cartilage and bone density of mushroom-shaped, photooxidized, osteochondral transplants: an experimental study on graft performance in sheep using transplants originating from different species
Source: BMC Musculoskelet Disord. 2005 Dec 15;6:60. doi: 10.1186/1471-2474-6-60 (PMC1343563; doi:10.1186/1471-2474-6-60)
Supplement: Additional File 2 — Semi-quantitative score system for histological assessment of synovial membrane sections. High numbers represent bad, low numbers good results. [file 1471-2474-6-60-S2.pdf]

Tab.2: Evaluation of synovial membranes

Score	Shape of synoviocytes	Proliferation of synovial layer	Neutrophiles	Eosinophiles	Lymphocytes	Plasmacells	Macrophages	Proliferation of vessels	Fibrin-Exsudation	Fibrotic metaplasia
0	normal	1-2 layers	none	none	none	none	none	none	none	none
1	slightly cubical	3-4 layers	few	few	few	few	few	mild	mild	mild
2	cubical	4-6 layers	moderate	moderate	moderate	moderate	moderate	moderate	moderate	moderate
3	prismatic	> 6 layers	everywhere	severe	severe	severe	everywhere	severe	excessive	severe
