# Supplementary material for: The effect of cartilage and bone density of mushroom-shaped, photooxidized, osteochondral transplants: an experimental study on graft performance in sheep using transplants originating from different species
Source: BMC Musculoskelet Disord. 2005 Dec 15;6:60. doi: 10.1186/1471-2474-6-60 (PMC1343563; doi:10.1186/1471-2474-6-60)
Supplement: Additional File 3 — Semi-quantitative score system for histological assessment of cartilage sections. High numbers represent bad, low numbers good results. [file 1471-2474-6-60-S3.pdf]

Tab.3 : Evaluation cartilage layer of osteochondral mushroom grafts and host matrix

Rating	Score Typ	0	1	2	3
Cartilage surface (graft)	a	smooth	even, but small rough areas	multiple rough areas	extremely rough
Pannus at surface (graft)	a	none	layer formation at small areas	continuous thin layer	continuous thick layer
Cartilage metachromasie (graft)	b	everywhere	mostly seen	small areas only	none
Collagen breakdown (graft)	a	none	1 small area	multiple small areas	visible everywhere
Fibrillation of matrix (graft)	a	none	1 small area	multiple small areas	visible everywhere
Dislocation of graft	a	none	1 side only (not excessive)	both sides (not excessive)	1 or both sides (severely)
Cleft formation in matrix	a	none	1 small cleft	1 big or 2 small clefts	> 3 clefts
Cartilage viability (graft)	b	everywhere	just top and bottom part	top or bottom part only	none
Cartilage viability (host)	b	everywhere	just top and bottom part	top or bottom part only	none
Chondrocyte proliferation (graft)	b	everywhere	mostly seen	small areas only	none
Chondrocyte proliferation (host)	b	everywhere	mostly seen	small areas only	none
Cluster formation (graft)	a	none	1 cluster	2-4 clusters	> 5 clusters
Cluster formation (host)	a	none	1 cluster	2-4 clusters	> 5 clusters
Graft-Host-Junction (fusion)	b	fusion	mostly fused	fusion at small areas only	none
Osteoclasts (graft)	a	none	1 per powerfield	2-4 per powerfield	> 5 osteoclasts per powerfield
Osteoclasts (bone)	a	none	1 per powerfield	2-4 per powerfield	> 5 osteoclasts per powerfield
Remodeling tide line/calcified cartilage	a	none	1/3 of the length	2/3 of the length	everywhere
Cutting cones through tide line/calcified cartilage	a	none	1 cone	2-4 cones	> 5 cones
Multinuclear cells below calcified layer	a	none	1 small area	multiple small areas	visible everywhere
