# Supplementary material for: The effect of cartilage and bone density of mushroom-shaped, photooxidized, osteochondral transplants: an experimental study on graft performance in sheep using transplants originating from different species
Source: BMC Musculoskelet Disord. 2005 Dec 15;6:60. doi: 10.1186/1471-2474-6-60 (PMC1343563; doi:10.1186/1471-2474-6-60)
Supplement: Additional File 4 — Results of macroscopic evaluation of grafts at the time of sacrifice (6 months). Overall, the equine (EN) together with the old bovine (BO) grafts retained the blue color the most, and only 2 grafts were partially overgrown by pannus-like tissue. Only 1/8 equine grafts was slightly sunk into the original cartilage defect. [file 1471-2474-6-60-S4.pdf]

Tab.4 : Observations after sample harvesting

Material	Number of condyles	Still blue color		Partially overgrown		Sunk in	
		lat	med	lat	med	lat	med
BN	16	8	6	4	8	6	12
BO	8	4	4	0	2	2	3
ON	8	3	3	1	2	1	3
OO	8	2	2	2	4	1	3
EN	8	4	4	1	1	1	0
HN	8	4	4	0	1	1	1
HO	8	2	0	3	4	2	4
Total	64	27	23	11	22	14	26
