# Supplementary material for: The effect of cartilage and bone density of mushroom-shaped, photooxidized, osteochondral transplants: an experimental study on graft performance in sheep using transplants originating from different species
Source: BMC Musculoskelet Disord. 2005 Dec 15;6:60. doi: 10.1186/1471-2474-6-60 (PMC1343563; doi:10.1186/1471-2474-6-60)
Supplement: Additional File 5 — Radiographic evaluation of osteochondral transplants at the time of sacrifice (6 months). [file 1471-2474-6-60-S5.pdf]

Tab.5 : Number of cysts radiographs

Material	Number of condyles	Cysts		Total
		lat	med	
BN	16	4	6	10 (62.5%)
BO	8	2	2	4 (50%)
ON	8	3	4	7 (25%)
OO	8	1	1	2 (25%)
EN	8	1	1	2 (25%)
HN	8	2	0	2 (25%)
HO	8	4	4	8 (100%)
Total	64	17	18	28
