# Supplementary material for: The effect of cartilage and bone density of mushroom-shaped, photooxidized, osteochondral transplants: an experimental study on graft performance in sheep using transplants originating from different species
Source: BMC Musculoskelet Disord. 2005 Dec 15;6:60. doi: 10.1186/1471-2474-6-60 (PMC1343563; doi:10.1186/1471-2474-6-60)
Supplement: Additional File 6 — Results of semi-quantitative histological evaluation of synovial membrane samples. Scores for a) degenerative aspects: 0 = none, 1 = mild, 2 = moderate, 3 = severe and b) regenerative aspects: 0 = good, 1 = medium, 2 = few, 3 = none. Note that most of the membranes are within physiological limits and only those of the human transplants show immunogenic reactions. [file 1471-2474-6-60-S6.pdf]

Tab.6: Overview of statistical results (synovial membranes)

Synovial Membran Score	Score	BN (n=32)	BO (n=16)	EN (n=16)	HN (n=16)	HO (n=16)	ON (n=16)	OO (n=16)	Overall Interaction
Shape Synoviocytes	a	0.31 ± 0.6	0	0	0.75 ± 0.7	0.88 ± 0.3	0.63 ± 0.5	0.38 ± 0.5	P<.0001
Proliferation Synoviocytes	a	0.19 ± 0.5	0	0	0.38 ± 0.5	0.13 ± 0.3	0.13 ± 0.3	0	P=.0354
Neutrophil Granulocytes	a	0	0	0	0	0	0	0	
Eosinophil Granulocytes	a	0	0	0	0	0	0.5	0	P=.0005
Lymphocytes	a	0.19 ± 0.4	0	0	1.75 ± 1	1 ± 0.73	0.38 ± 0.5	0	P<.0001
Plasmacells	a	0.12 ± 0.3	0	0	0.88 ± 0.62	0.5 ± 0.5	0.5 ± 0.5	0	P<.0001
Macrophages	a	0.12 ± 0.3	0	0	0.75 ± 0.5	0.25 ± 0.5	0.13 ± 0.3	0	P<.0001
Bloodvessels	a	0	0	0	0	0	0	0	
Fibrinous Exsudate	a	0.19 ± 0.4	0.31 ± 0.4	0	0.375 ± 05	0.25 ± 0.4	0.25 ± 0.4	0	P<.0393
Fibrotic Metaplasia	a	0	0	0	0	0	0	0	

a. for degenerative aspects: 0=none, 1=mild, 2=moderate, 3=severe

b. for regenerativ aspects: 0=good, 1=medium, 2=few, 3=none

Low scores represent good results, while high scores mean less good results
