# Supplementary material for: The effect of cartilage and bone density of mushroom-shaped, photooxidized, osteochondral transplants: an experimental study on graft performance in sheep using transplants originating from different species
Source: BMC Musculoskelet Disord. 2005 Dec 15;6:60. doi: 10.1186/1471-2474-6-60 (PMC1343563; doi:10.1186/1471-2474-6-60)
Supplement: Additional File 7 — Overview of statistical results (cartilage and subchondral bone) and results of semi-quantitative histological evaluation of cartilage samples. The equine group (EN) accumulates most of the best ( = lowest scores) and second best scores. Most of the lowest scores were found in the human group according to the old process (HO). Scores for a) degenerative aspects: 0 = none, 1 = mild, 2 = moderate, 3 = severe and b) regenerative aspects: 0 = good, 1 = medium, 2 = few, 3 = none. Low scores represent good results, while high scores mean less good results. Colors indicate performance best group second best group worst group [file 1471-2474-6-60-S7.pdf]

Tab.7: Overview of statistical results (cartilage and subchondral bone)

Cartilage Score	Score	BN (n=32)	BO (n=16)	EN (n=16)	HN (n=16)	HO (n=16)	ON (n=16)	OO (n=16)	Overall Interaction	Individual interaction
Cartilage Surface	a	1.71 ± 0.8	1.26 ± 0.6	0.44 ± 0.5	1.78 ± 1.3	1.75 ± 1.5	1.58 ± 1	1.2 ± 1.4	p=.0017	bn,en: P<.0001;bo,en: P=.0191; en,hn: P=.0002; en,hn: P=.0167; en,on: P=.0024; en,oo: P=.0301
Pannus surface	a	1.77 ± 1.2	1.32 ± 1	0.6 ± 1	0.37 ± 0.7	2.43 ± 0.9	0.58 ± 1	1.25 ± 1.3	p<.0001	bn,en ; P<.0001; bo,en: P=.0007; en,ho: P<.0001; en,oo: P=.0012; hn,ho: P<.0001
Cartilage metachromasie	b	1.57 ± 1.1	1.12 ± 0.8	1.38 ± 0.5	1.43 ± 0.7	1.3 ± 1.2	2.17 ± 0.7	1.67 ± 0.7	p=.0935	bn,on: P=.0485; bo,on: P=.0025; en,on: P=.0203; hn,on: P=.0353; ho,on: P=.0340
Collagen breakdown	a	1.47 ± 1.1	0.56 ± 0.6	1.25 ± 0.6	0.33 ± 0.5	0.5 ± 0.9	0.83 ± 0.84	0.47 ± 0.6	p<.0001	bn,bo: P=.0004; bn,hn: P<.0001; bn,ho: P=.0030; bn,ho: P=.0223; bn,oo: P=.0001; bo,en: P=.0167; en,hn: P=.0019; en,ho: P=.0326; hn,oo: P=.0075
Fibrillation of matrix	a	0.63 ± 0.8	0.5 ± 0.5	0.25 ± 0.5	0.46 ± 0.7	1 ± 0	0.83 ± 1.2	0.2 ± 0.4	p=.0976	en,on: P=.0273; ho,oo: P=.0396; on,oo. P=.0184
Dislocation of graft	a	2.18 ± 0.9	1.63 ± 1.0	0.36 ± 0.9	0.5 ± 0.9	2.57 ± 0.8	1.38 ± 1.3	1 ± 1.3	P<.0001	
Cleft formation in matrix	a	0.73 ± 0.9	0.56 ± 0.9	0.5 ± 0.73	1.57 ± 1.5	2.28 ± 1.1	0.67 ± 1.15	0.47 ± 0.7	P=.0003	bn,hn: P=.0110, bn,ho: P=.0004; bo,hn: P=.0002; en,hn: P=.0042,en,ho: P=.0001; hn,on: P=.0235; hn,oo: P=.0009; ho,oo: P=.0001
Cartilage viability (graft)	b	1.57 ± 1.1	1.25 ± 0.8	1.38 ± 0.5	1.43 ± 0.8	1.63 ± 1.2	2 ± 0.9	1.56 ± 0.8	P=.3360	bo,on: P=.0150
Cartilage viability (host)	b	0.72 ± 0.6	0.75 ± 0.5	0.25 ± 0.5	0.6 ± 0.6	0.62 ± 0.7	0.67 ± 0.5	0.5 ± 0.7	P=.2091	bn,en: P=.0180; bo,en: P=.0183
Chondrozytenprolif.(graft)	b	2.37 ± 1	1.75 ± 0.8	1.88 ± 0.7	2.2 ± 0.6	2.67 ± 0.5	1.75 ± 1.1	1.67 ± 0.9	P=.0150	bn,bo: P=.0208; bn,on: P=.0358; bn,oo: P=.0104; bo,ho: P=.0109; en,ho: P=.0273; ho,on: P=.0160; ho,oo: P0.0062
Chondrozytenprolif. (host)	b	0.91 ± 0.6	0.81 ± 0.4	0.5 ± 0.5	0.6 ± 0.5	0.57 ± 0.8	0.75 ± 0.6	0.88 ± 1	P=.3527	bn,en: P=.0496
Cluster formation (graft)	a	0.3 ± 0.2	0	0	0	0	0	0	P=.8571	
Cluster formation (host)	a	2 ± 0.8	1.5 ± 0.7	0.75 ± 0.7	1.3 ± 1.1	2.18 ± 0.8	1.58 ± 0.9	1.63 ± 0.8	P<.0001	bn,en: <.0001; bn,hn: P=.0090; bo,hn: P=.0134; bo,ho: P=.0231; en,ho: P<.0001; en,on: P=.0110; en,oo: P=.0041; hn,ho: P=.0041
Fusion	b	2.8 ± 05	2.56 ± 1	2.19 ± 0.5	2.87 ± 0.4	2 ± 1.5	2.08 ± 0.9	2.12 ± 1.1	P=.0065	bn,en: P=.0143; bn,ho: P=.0264; bn,on: P=.0095: bn,oo: P=.0071; en,hn: P=.0191; hn,ho: P=.0259; hn,on: P=.0123; hn,oo: P=.0107
Osteoclast host	a	0.56 ± 0.7	0.38 ± 0.5	0.25 ± 0.5	0.50 ±0.8	0.25 ± 0.5	0.63 ± 0.5	0.38 ± 0,72	P= .45	
Osteoclasts graft	a	0.19 ± 0.4	0.38 ± 0.7	0.25 ± 0.5	0.13 ± 0.3	0.38 ± 0.5	0.13 ± 0.3	0	P=.1458	bo,on: P=.0162; ho,oo: P=.0162
Remodeling tide line/calcified cartilage	a	2.58 ± 0.9	1.5 ± 1.3	0.63 ± 1.1	0.25 ± 0.5	1 ± 1.0	1.57 ± 1.1	2 ± 0.9	P=<.0001	bn,bo: P=.0009; bn,en: P<.0001; bn,hn: P<.0001; bn,ho: P<.0001; bn,on: P=.0029; bo,en: P=.0136; bo,hn: P=.0005; en,on: P=.0100; en,oo: P=.0001; hn,ho: P=.0337; hn,on: P=.0004; hn,oo: P<.0001; ho,oo: P=.0050
Cutting cones through tide line/calcified cartilage	a	1 ± 1.3	0.5 ± 0.5	0.75 ± 1.1	1.13 ± 1.1	0.71 ± 0.7	1.2 ± 0.4	0.83 ± 0.7	P=.4850	
multinuclear cells below calcified layer	a	1.5 ± 1.2	0.33 ± 0.8	0.38 ± 1.0	0	0.29 ± 0.7	0.4 ± 0.8	0.3 ± 0.8	P=.0068	bn,bo: P=.0021; bn,en: P=.0018; bn,hn: P<.0010; bn,huo: P=.0010; bn,on: P=.0050; bn,oo: P=.0021

a. for degenerative aspects: 0=none, 1=mild, 2=moderate, 3=severe
b. for regenerativ aspects: 0=good, 1=medium, 2=few, 3=none
Low scores presents good results, while high scores mean less good results

best group
second best group
worst group
