# Supplementary material for: The effect of cartilage and bone density of mushroom-shaped, photooxidized, osteochondral transplants: an experimental study on graft performance in sheep using transplants originating from different species
Source: BMC Musculoskelet Disord. 2005 Dec 15;6:60. doi: 10.1186/1471-2474-6-60 (PMC1343563; doi:10.1186/1471-2474-6-60)
Supplement: Additional File 8 — Results of the histomorphometrical measurements. The equine group (EN) showed the highest percentage of bone and the least amount of cystic lesions. [file 1471-2474-6-60-S8.pdf]

Tab.8 : Overview of statistical results (histomorphometry)

Material	BN	BO	EN	HN	HO	ON	OO	P- Value overall	P-Value individual
Fibrous tissue	37.06 ± 8.4	35.87 ± 10.6	29.87 ± 5.6	31.97 ± 7.8	48.66 ± 11.4	33.86 ± 8.8	37.94 ± 9.1	P=.0043	bn,ho: P=.0041; bo,ho: P=.0059; en,ho: P=.0002; hn,ho: P=.0004; ho,on: P=.0017; ho,oo: P=.0243
Bone	44.02 ± 7.1	43.41 ± 9.5	51.31 ± 11.1	44.85 ± 8.6	30.82 ± 4.6	45.53 ± 8.0	49.20 ± 3.9	P=.0002	bn,en: P=.0429; bn,ho: P=.0002; bo,ho: P=.0020; en,ho: P<.0001; hn,ho: P=.0007; ho,on: P=.0004; ho,oo: P<.0001
Cartilage	7.90 ± 3.9	11.0 ± 3.42	9.66 ± 3.9	13.86 ± 3.5	11.61 ± 5.7	7.13 ± 4.8	6.00 ± 3.3	P=.0043	bn,hn: P=.0017; bn,ho: P=.0446; bo,oo: P=.0241; hn,on: P=.0021; hn,oo: P=.0006; ho,on: P=.0363; ho,oo: P=.0019
